# Clinical features, risk of mass enlargement, and development of endocrine hyperfunction in patients with adrenal incidentalomas: a long-term follow-up study

**DOI:** 10.1007/s12020-020-02476-1

**Published:** 2020-09-11

**Authors:** Pierpaolo Falcetta, Francesca Orsolini, Elena Benelli, Patrizia Agretti, Paolo Vitti, Caterina Di Cosmo, Massimo Tonacchera

**Affiliations:** 1 Section of Endocrinology, Department of Clinical and Experimental Medicine, University Hospital of Pisa, Pisa, Italy; 2 Laboratory of Chemistry and Endocrinology, University Hospital of Pisa, Pisa, Italy

**Keywords:** Adrenal incidentaloma, AI, Autonomous cortisol secretion, ACS, Adrenal, Cushing’s syndrome

## Abstract

**Purpose:**

To evaluate the risk of mass enlargement and endocrine function modification in patients with adrenal incidentaloma (AI).

**Methods:**

In this retrospective study, we examined clinical and hormonal characteristics of 310 patients with AI (200 females and 110 males; age: 58.3 ± 12.9 years), followed up for a median (interquartile range) of 31.4 months (13.0–78.6) and evaluated for possible modification in adrenal mass size and hormonal function. The hormonal evaluation included morning serum cortisol and plasma ACTH at 8 a.m., aldosterone, plasma renin activity/direct renin concentration, and 24-h urine metanephrines/normetanephrines. One microgram overnight dexamethasone suppression test (DST) was performed. Autonomous cortisol secretion (ACS) was diagnosed in the presence of cortisol after 1 mg DST > 5 μg/dl (138 nmol/l) or >1.8 and ≤5 μg/dl (50–138 nmol/l) and at least one of the following: (i) low ACTH; (ii) increased 24-h urinary-free cortisol; (iii) absence of cortisol rhythm; and (iv) post-LDDST cortisol level > 1.8 μg/dl (50 nmol/l). When there was no biochemical evidence of adrenal hormonal hyperactivity, AIs were classified as nonfunctioning (NFAIs). The mass was considered significantly enlarged when the size increase was more than 20% and at least 5 mm compared to baseline.

**Results:**

At diagnosis, NFAIs were found in 209 patients, while ACS and overt adrenal hyperfunction were diagnosed in 81 and 20 patients, respectively. During follow-up, 3.3% and 1.5% of patients with NFAI developed subtle and overt endocrine hyperfunction, respectively, while a significant mass enlargement was observed in 17.7% of all AIs. The risk of developing ACS was significantly higher in patients with adenoma >28 mm (hazard ratio [HR] 12.4; 95% confidence interval [CI], 2.33–66.52, *P* = 0.003), in those with bilateral adrenal tumors (HR: 5.36; 95% CI, 1.17–24.48, *P* = 0.030), and with low/suppressed ACTH values (HR: 11.2, 95% CI 2.06–60.77; *P* = 0.005). The risk of mass enlargement was lower for patients in the fourth quartile of body mass index than those in the first quartile (HR 0.33; 95% CI, 0.14–0.78; *P* = 0.012).

**Conclusions:**

In patients with AI, the risk of developing hormonal hyperfunction and mass enlargement is overall low, although some tumor characteristics and anthropometric features might increase this risk. Taking account of all these aspects is important for planning a tailored follow-up in AI patients.

## Introduction

Adrenal incidentalomas (AIs), which define clinically unapparent adrenal masses discovered by abdominal imaging procedures for unrelated reasons, have become a common finding in clinical practice, ranging between 1 and 7% in different series [1–6]. Whereas most AIs are benign nonfunctioning AIs (NFAIs), about 5–30% are associated with some degree of hormone production [7–10]. The most common endocrine abnormality in patients bearing an AI is the so-called autonomous cortisol secretion (ACS). ACS is characterized by subtle cortisol excess due to autonomous cortisol-secreting adenomas, in the absence of signs or symptoms of overt hypercortisolism [8]. In recent years, this condition has become a topic of growing interest. Indeed, it is fairly well known that ACS, although asymptomatic, is associated with increased metabolic derangement and mortality, mainly due to cardiovascular events [11–14]. The right timing of follow-up in NFAIs is still unclear [15], since data on the natural history and possible changes in adrenal size and hormonal pattern are controversial. In this retrospective study, we have evaluated the cumulative risk of mass enlargement and development of endocrine abnormalities over time in patients with AIs.

## Patients and methods

We retrospectively analyzed the records of 310 consecutive patients with diagnosed AI by abdominal ultrasonography (US) (31%), computed tomography (CT) (61%), or MRI (8%) and referred to the Endocrine Unit of University Hospital of Pisa, Italy, between January 2010 and September 2019. Data were anonymously collected and analyzed from clinical records. Ethical approval was waived by the local Ethics Committee of University of Pisa in view of the retrospective nature of the study and all the procedures being performed were part of the routine care. We excluded patients with any condition or taking any drug known to interfere with steroid hormone secretion or metabolism. We also excluded patients who underwent radiological investigation for suspected cancer and those with adrenal masses <1 cm. All AIs diagnosed by abdominal US were confirmed by unenhanced CT scan and followed up with the same imaging procedure over time. When bilateral adenomas were found, the diameter of the largest adenoma was reported. All the patients underwent a complete hormonal evaluation, which included morning serum cortisol and plasma ACTH at 8 a.m., 24-h urine metanephrines and normetanephrines, and 1 mg dexamethasone suppression test (DST, dexamethasone 1 mg p.o. at midnight). In hypertensive patients, aldosterone and plasma renin activity (PRA) or direct renin concentration (DRC) were measured. AIs were defined as nonfunctioning when there was no biochemical evidence of adrenal hormonal hyperactivity. When endocrine abnormalities were found, confirmatory tests were always performed: 48-h—2 mg/day DST (low dose DST (LDDST), i.e., dexamethasone 0.5 mg p.o. every 6 h for 2 days) for suspected ACS [16], saline loading test or captopril test for suspicion of primary hyperaldosteronism (PHA), and iodine-123-metaiodobenzylguanidine scintigraphy for suspected pheochromocytoma. Following the currently available guidelines [17, 18], the diagnosis of ACS was based on a post-DST cortisol level >5 μg/dl (138 nmol/l) or >1.8 and ≤5 μg/dl (50–138 nmol/l, possible cortisol secretion) combined with an abnormal result in at least one of the following tests to evaluate HPA axis: (i) high urinary-free cortisol (UFC) values (≥405 μg/24 h (1117.8 mmol/24 h); (ii) absence of cortisol rhythm (midnight serum cortisol > 7.5 μg/dl (220 nmol/l); (iii) low ACTH levels (<10 pg/ml (2.2 pmol/l); and (iv) post-LDDST cortisol level > 1.8 μg/dl (50 nmol/l), in the absence of clinical signs or symptoms of cortisol excess. When “possible cortisol secretion” was found without any of the additional features of ACS previously mentioned, the AI was classified as nonfunctioning. In the clinical examination, weight, height, body mass index (BMI), systolic blood pressure, and diastolic blood pressure were measured by using standard methods. Fasting plasma glucose, glycated hemoglobin, serum electrolytes, and lipids were evaluated by routine laboratory methods. After the initial diagnosis, a complete hormonal and radiological assessment was repeated at 6 and 12 months and then at 1-year intervals. At diagnosis and during the follow-up period, all patients maintained their usual diet, and patients with hypertension were switched to calcium antagonists at least 2 weeks before the hormonal evaluation. The AI was defined as enlarged when the increase of the maximum diameter was more than 20% and at least 5 mm compared to baseline measurement. Hypertension was defined as blood pressure ≥ 140/90 mmHg or current use of antihypertensive medications [19]. Diabetes mellitus, impaired fasting glucose (IFG), and impaired glucose tolerance were defined using American Diabetes Association criteria [20]. Dyslipidemia was defined in the presence of total cholesterol levels ≥200 mg/dl (5.17 mmol/l), LDL-C > 150 mg/dl (3.88 mmol/l), or HDL-C ≤ 50 mg/dl (1.3 mmol/l) for females and ≤40 mg/dl (1 mmol/l) for males, triglycerides ≥ 150 mg/dl (1.69 mmol/l), or use of lipid-lowering medications. Data about cerebrovascular (stroke or transient ischemic attack), cardiovascular disease (angina pectoris, myocardial infarction, or revascularization procedures), and atherosclerosis (hemodynamically significant carotid and/or lower limb arteries stenosis) were collected. Thirteen patients (mean age 52 ± 14 years; mean AI size 38 ± 15 mm) underwent adrenalectomy: two with NFAIs, three with ACS, five with Cushing’s syndrome (CS), one of which was diagnosed as having adrenocortical carcinoma, one with hyperaldosteronism, and two with pheochromocytoma. Observation for these patients was censored at the time of surgery [median follow-up 14.1 months (IQR, 9.8–38.3 months)].

### Laboratory assays

All the hormonal determinations were performed in the same laboratory through the years of the study, using kits from the same companies. Immunometric serum cortisol and UFC measurements were performed performed by Access Cortisol assay, a competitive binding immunoenzymatic assay, on Beckman Coulter UniCel DxI 600 automated platforms (Beckman Coulter Diagnostics, Brea, CA, USA). Plasmatic ACTH was measured by Immulite 2000 ACTH (Siemens, Healthcare Diagnostics Products, GmbH Marburg, Germany). Plasmatic aldosterone and DRC were respectively measured by Liaison Aldosterone and Liaison Direct Renin using Liaison XL automated platform (DiaSorin SpA, Saluggia, Italy). PRA was measured by RIA methods (PRA DiaSorin GammaCoat, DiaSorin SpA, Saluggia, Italy) and urinary metanephrine and normetanephrine were measured by MetCombi Plasma ELISA Metanephrine/Normetanephrine (IBL International GmbH Hamburg, Germany). Inter-assay and intra-assay coefficient of variation of analytes were, respectively, cortisol from 6.4 to 7.9% and from 4.4 to 6.7%, ACTH from 6.1 to 10% and from 6.7 to 9.5%, aldosterone from 5.8 to 10.5% and from 2.1 to 4.2%, DRC from 0.6 to 2.7% and from 1.2 to 3.7%, PRA from 5.6 to 7.6% and from 4.6 to 10%, urine metanephrine from 5.4 to 7.6% and from 5.8 to 8.6% and normetanephrine from 6.7 to 13.4% and from 6.3 to 9.9%, and UFC from 6 to 7.9% and from 5.5 to 6.7%.

### Statistical analysis

Variables were preliminarily tested for normal distribution with the Shapiro–Wilk test and data were expressed as mean ± SD, or median (IQR), as appropriate. Continuous variables were compared by the Student’s *t* test assuming equal or nonequal variance with the Levene’s test when normally distributed or Mann–Whitney *U* test when nonnormally distributed. Categorical variables were compared by the chi-square test or Fisher’s exact tests. Logistic regression analysis was performed to assess the relationship between anthropometric, demographic, biochemical data, and presence of ACS at diagnosis. Kaplan–Meier curves with Cox proportional hazard model analysis were used to estimate the cumulative risk of mass enlargement or ACS occurrence during follow-up, after adjusting for several confounding factors. The criteria of censoring in Kaplan–Meier analysis were the date of death/surgery or the last follow-up of the patient. To assess the cut-off of AI size with the best sensitivity (SN) and specificity (SP) for detecting NFAI patients at risk for developing ACS over time, the receiver operating characteristic (ROC) analysis was performed. The level of statistical significance was set at *P* less than 0.05. All statistical analyses were performed using SPSS (IBM SPSS Statistics, version 25).

## Results

A total of 310 patients (200 females and 110 males) with a mean ± SD age of 58.3 ± 12.6 years (range: 17–85 years) were selected for this study. Sixty-two patients (20%) had bilateral adrenal lesions. At diagnosis, 67% (209 out of 310) had NFAI, while 20 had an overt endocrine function: 9 (2.9%) with CS, 9 (2.9%) with hyperaldosteronism, and 2 (0.7%) with pheochromocytoma. A total of 83 subjects (26.8%) showed cortisol values after 1 mg DST between 1.9 and 5 μg/dl (50–138 nmol/l) (possible cortisol secretion) and 11 (3.5%) displayed cortisol concentrations after 1 mg DST > 5 μg/dl (138 nmol/l). Overall, 81 subjects (26.1%) were diagnosed as having ACS. Mean density of AIs was 15 ± 7 Hounsfield units (HU). General characteristics of the entire cohort are reported in Table 1. Comparing NFAI with ACS patients (Table 1), no significant differences were found in sex distribution, BMI, and UFC. In contrast, ACS patients were significantly older (62 ± 12.8 vs 57.3 ± 12.1 years, *P* = 0.004) and had a bigger adrenal mass size (mean size: 27.1 ± 9.8 vs 19.9 ± 8.5 mm, *P* < 0.0001) compared to NFAI. As expected, basal and post 1 mg DST serum cortisol was significantly higher, and ACTH was lower in the ACS group than in the NFAI group. Bilaterally located AIs were more frequently encountered in patients with ACS compared to those with NFAI (33.3 vs 14.8%, *P* < 0.0001). No significant differences were found in obesity prevalence or presence of glucose intolerance, while a significantly higher percentage of patients with ACS were hypertensive (65.4 vs 47.4%, *P* = 0.006). In a multiple regression model, including also age, sex, BMI, size, and bilaterality of adenomas, the presence of hypertension was significantly associated with ACS (OR 1.95, *P* = 0.045), with an independent role for age (OR 1.05, *P* < 0.0001), and BMI (OR 1.11, *P* < 0.0001), and independently from sex and adenoma size. The diagnosis of ACS was associated with adenoma size (OR 1.08, *P* < 0.0001), bilateral adrenal lesions (OR 2.23, *P* = 0.017), and age (OR 1.03, *P* = 0.011), independently from sex and BMI (Table 2).

**Table 1:** Clinical and biochemical characteristics of the whole cohort and subjects with NFAI and ACS at diagnosis

	All AIs (*n* = 310)	NFAI (*n* = 209)	ACS (*n* = 81)	*P* value NFAI vs. ACS
Males	110 (35.5)	77 (36.8)	24 (29.6)	0.247
Age, years	58.3 (12.6)	57.3 (12.1)	62.0 (12.8)	0.004
BMI, kg/m^2^	28.5 (5.7)	28.8 (6.1)	27.9 (5.0)	0.288
Cortisol, μg/dl	10.9 (8.1–14.4)	9.9 (7.4–13.8)	11.7 (9.4–15.0)	0.001
ACTH, pg/ml	12 (6–19)	13 (8–19)	7 (5–12)	<0.0001
Cortisol post 1 mg DST, μg/dl	1.4 (0.9–2.3)	1.1 (0.8–1.4)	2.7 (2.3–3.6)	<0.0001
UFC, μg/24 h	143 (83–213)	127.5 (98.2–214.2)	150 (75.5–215)	0.939
Adenoma size, mm	22.1 (9.8)	19.9 (8.5)	27.1 (9.8)	<0.0001
Bilateral AIs	62 (20)	31 (14.8)	27 (33.3)	<0.0001
Smoking habit				
Never	197 (63.5)	134 (64.1)	50 (61.7)	0.908
Current	56 (18.1)	35 (16.7)	17 (21)	
Former	57 (18.4)	40 (19.2)	24 (29.6)	
Obesity classes				
Overweight	109 (35.2)	66 (31.6)	31 (38.3)	0.278
Class 1	70 (22.6)	49 (23.4)	18 (22.2)	0.825
Class 2	18 (5.8)	14 (6.7)	4 (4.9)	0.577
Class 3	14 (4.5)	12 (5.7)	2 (2.5)	0.363
Hypertension	169 (54.5)	99 (47.4)	53 (65.4)	0.006
Diabetes	60 (19.4)	39 (18.7)	14 (17.3)	0.786
IFG	28 (9.0)	17 (8.1)	11 (13.6)	0.159
IGT	12 (3.9)	6 (2.9)	4 (4.9)	0.473
Dyslipidemia	141 (45.5)	90 (43.1)	38 (46.9)	0.553
Stroke	8 (2.6)	5 (2.4)	1 (1.2)	0.534
MI	9 (2.9)	7 (3.3)	1 (1.2)	0.450
ATS	11 (3.5)	5 (2.4)	4 (4.9)	0.272
AH use	156 (50.3)	91 (43.5)	50 (61.7)	0.006
AHA use	46 (14.8)	34 (16.3)	7 (8.6)	0.132
Statin use	56 (18.1)	34 (16.3)	16 (19.8)	0.491

Data are expressed as mean (SD), median (IQR), or absolute number (percentage). SI conversion factors: cortisol, ×27.59; ACTH, ×0.22; UFC, ×2.76; total and HDL cholesterol, ×0.026; triglicerides, ×0.011; and FPG, ×0.06

*ACS* autonomous cortisol secretion, *AH* antihypertensive, *AHA* antihyperglycemic agent, *ATS* atherosclerosis, *DBP* diastolic blood pressure, *FPG* fasting plasma glucose, *HDL-C* high-density lipoprotein cholesterol, *IFG* impaired fasting glucose, *IGT* impaired glucose tolerance, *LDL-C* low-density lipoprotein cholesterol, *MI* myocardial infarction, *NFAI* nonfunctioning adrenal incidentaloma, *SBP* systolic blood pressure, *UFC* urinary-free cortisol

**Table 2:** Multivariable logistic regression analysis for predictors of arterial hypertension and ACS in the entire cohort

	AH			ACS		
	OR	95% CI	*P* value	OR	95% CI	*P* value
Male sex	1.60	1.01–2.74	0.082	0.65	0.35–1.20	0.170
Age, 1 year	1.05	1.02–1.07	<0.0001	1.03	1.01–1.06	0.011
BMI, 1 kg/m^2^	1.11	1.06–1.16	<0.0001	0.98	0.93–1.03	0.473
Adenoma size, 1 mm	1.01	0.99–1.04	0.506	1.08	1.05–1.12	<0.0001
ACS	1.95	1.01–3.47	0.045	–	–	–
Bilateral AI	0.81	0.42–1.56	0.535	2.23	1.15–4.32	0.017

*ACS* autonomous cortisol secretion, *AH* arterial hypertension, *AI* adrenal incidentaloma, *BMI* body mass index

### Risk of endocrine function development or modification during follow-up

**Fig. 1:**
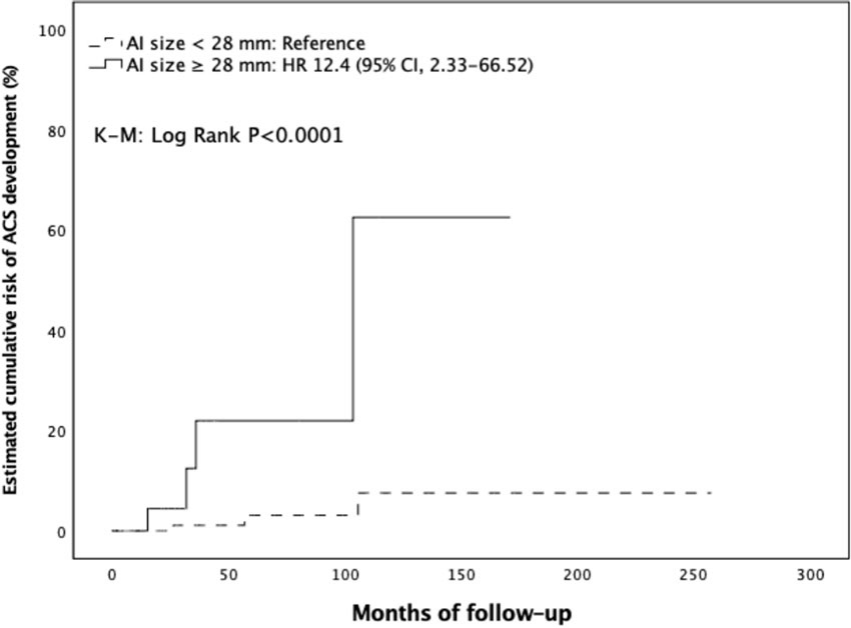
Estimated cumulative risk of ACS development during follow-up in patients according to baseline adenoma size

**Fig. 2:**
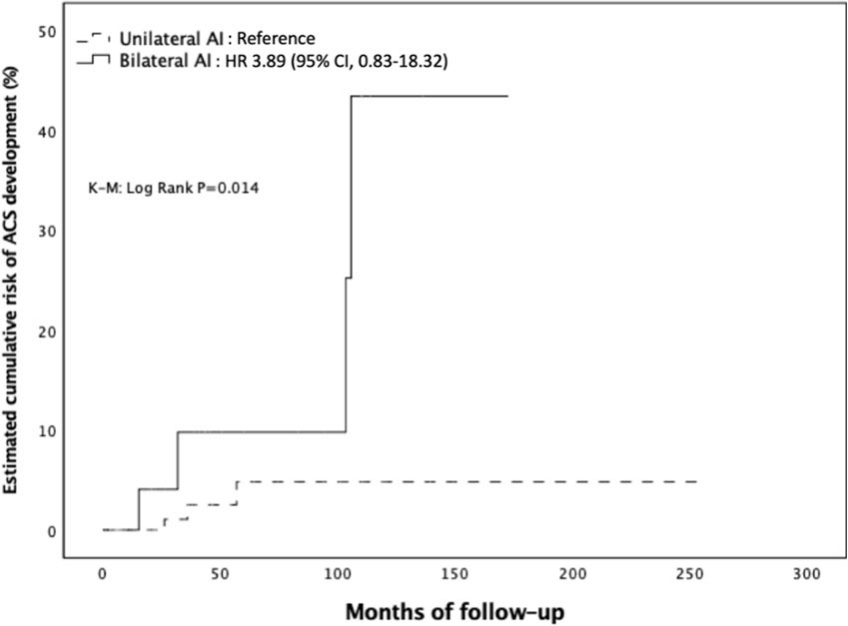
Estimated cumulative risk of ACS development during follow-up in patients according to adenoma laterality

**Fig. 3:**
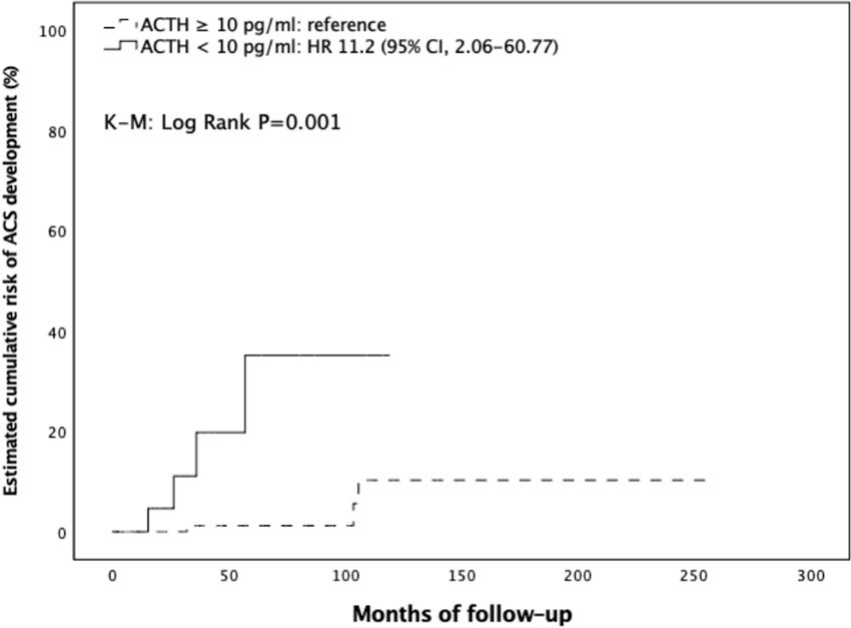
Estimated cumulative risk of ACS development during follow-up in patients according to ACTH values at diagnosis

The median follow-up duration was 31.4 months (IQR, 13.0–78.6 months). Overall, 10 patients out of 209 (4.8%) with NFAI at diagnosis developed endocrine hyperfunctioning during the follow-up period. Seven (3.3%) developed ACS, one (0.5%) developed PHA, while two (1%) developed pheochromocytoma. The general characteristics of the three patients with NFAI at the study entry who developed adrenal hyperfunction during follow-up are summarized in Table 3. Briefly, they were two females and one male, with a mean age of 62 ± 8 years and mass size of 3 ± 0.9 cm. Two out of three showed a significant mass enlargement during follow-up, while in one the AI size remained stable. None of the patients was taking drugs or medications known to interfere with the hormonal evaluation. Furthermore, 2 patients out of 81 (2.5%) with ACS at baseline developed overt CS during the follow-up, and 1 (1.2%) developed a concomitant aldosterone secretion. When considering only those developing overt hormone secretion, five patients out of six (83.3%) displayed a significant mass enlargement during follow-up, with three of them showing an increase above 10 mm. Cumulative risk of ACS development was 0.5%, 1.9%, and 2.4% at 2, 3, and 5 years, respectively. Comparing NFAI patients who developed ACS (ACSfu+, *n* = 7) with those showing no endocrine hyperfunction during follow-up (ACSfu−, *n* = 202), at the study entry, we found that adenoma size and prevalence of bilateral adenomas were significantly greater in ACSfu+ than ACSfu− patients, while ACTH values significantly lower in the former as compared to those in the ACSfu− group. Cortisol concentrations after 1 mg DST tended to be higher in ACSfu+ patients compared to those in ACSfu− group (1.6 (1.0–3.1) vs 1.1 (0.8–1.4) μg/dl, *P* = 0.079); however, no association between the degree of cortisol hypersecretion (cortisol levels post 1 mg DST) and metabolic and cardiovascular complications was found (data not shown). Finally, ACSfu+ patients had a higher prevalence of IFG, stroke, and atherosclerosis compared to those in ACSfu−group (Table 4). Using the ROC curve analysis, we explored the relationship between the adenoma size and the risk of newly diagnosed ACS over time. The analysis confirmed this association (AUC: 0.730, *P* = 0.039) and showed that the cut-off of size with the best compromise between SN and SP in predicting the development of ACS was set at 28 mm (SN, 57%; SP, 85%) (Supplementary Material S1). Using this threshold, we divided the entire cohort in two groups: those with adenoma size <28 mm (Group A) or ≥28 mm (Group B). The occurrence of ACS was significantly higher in Group B than Group A [3.48 per 1000 person-years (PYs) vs 0.39 per 1000 PYs; K–M log rank: chi-square 13.207; df: 1; *P* < 0.0001]. Adjusting for multiple confounders, we found that patients in Group B had more than 12-fold increased risk for developing ACS compared to those in Group A (hazard ratio (HR) 12.4; 95% confidence interval (CI), 2.33–66.52, *P* = 0.003) (Fig. 1) (Supplementary Material S2). Concerning the localization of adrenal mass, development of ACS was less frequent in patients with unilateral mass than those with bilaterally sided AIs (0.41 per 1000 PYs vs 2.48 per 1000 PYs; K–M log rank: chi-square 6.009; df: 1; *P* = 0.014) (Fig. 2), the latter being at higher risk for ACS development (HR: 5.36; 95% CI, 1.17–24.48, *P* = 0.030) even after adjusting for multiple confounders. However, when adding AI size to the model, this risk was no longer statistically significant (HR 3.89, 95% CI 0.83–18.32; *P* = 0.085) (Supplementary Material S3). Furthermore, when considering ACTH levels as a categorical variable [< or ≥10 ng/l (2.2 pmol/l)], patients in ACSfu+ group showed a significantly higher frequency of ACTH suppression compared to those in ACSfu− group (57.1 vs 12.1%, *P* = 0.007). The survival analysis showed a higher incidence of ACS in patients with ACTH < 10 ng/l compared to those with higher levels of ACTH (3.61 per 1000 PYs vs 0.38 per 1000 PYs; K–M log rank: chi-square 11.625; df: 1; *P* = 0.001) (Fig. 3). After adjusting for multiple confounders, the presence of low ACTH levels at entry was found to be an independent risk factor for ACS development during follow-up (HR: 11.2, 95% CI 2.06–60.77; *P* = 0.005) (Supplementary Material S4).

**Table 3:** Clinical, biochemical, and radiological characteristics of patients with NFAI at diagnosis who developed adrenal hyperfunction during follow-up

Age (years)/sex	AI size (cm)/HU/side	Clinical findings at diagnosis	Interfering drugs	Endocrine abnormality at follow-up	Time between diagnosis and endocrine hyperfunction onset	AI enlargement (cm)	New onset comorbidities
68/M	2.5/11/L	AH; O	No	PHA	2 years	N (2.5)	Worsened AH; Hypokalemia
53/F	2.4/<10/R	–	No	PHEO	6 years	Y (3.7)	T2DM
69/F	4.0/35/R	AH; T2DM; DL	No	PHEO	3 years	Y (5.3)	Worsened T2DM/AH; Takotsubo syndrome

*AI* adrenal incidentaloma, *AH* arterial hypertension, *DL* dyslipidemia, *HU* Hounsfield’s units, *L* left, *O* obesity, *PHA* primary hyperaldosteronism, *PHEO* pheochromocytoma, *R* right, *T2DM* type 2 diabetes mellitus

**Table 4:** Comparison of the clinical and biochemical characteristics at baseline between patients with and without mass enlargement during follow-up, and between patients developing and not developing ACS during follow-up

	AI enlargement− (*n* = 257)	AI enlargement+ (*n* = 53)	*P* value	ACSfu− (*n* = 202)	ACSfu+ (*n* = 7)	*P* value
Males	93 (36.2)	17 (32.1)	0.569	75 (37.1)	2 (28.6)	0.629
Age, years	58.9 (12.0)	55.6 (14.6)	0.127	56.9 (13.0)	57.0 (9.2)	0.986
BMI, kg/m^2^	28.8 (5.8)	26.8 (4.9)	0.022	28.8 (6.1)	28.6 (6.5)	0.959
Cortisol, μg/dl	10.4 (8.0–14.2)	12 (9.2–15.6)	0.073	9.9 (7.4–13.8)	10 (7.1–13.0)	0.640
ACTH, pg/ml	12 (6–19)	12 (5–19)	0.538	14 (8–20)	5 (5–8.7)	0.005
Adenoma size, mm	21.8 (9.5)	23.7 (11.3)	0.210	19.7 (8.3)	28.4 (10.0)	<0.001
Bilateral AIs	49 (19.1)	13 (24.5)	0.495	21 (12.2)	4 (57.1)	0.002
Smoking habit						
Never	167 (65)	30 (56.6)	0.944	131 (64.9)	3 (42.9)	0.580
Former	45 (17.5)	12 (22.7)		39 (19.3)	3 (42.9)	
Current	45 (17.5)	11 (20.7)		32 (15.8)	1 (14.2)	
Obesity class						
Overweight	94 (36.6)	15 (28.3)	0.251	63 (32)	1 (14.3)	0.728
Class 1	58 (22.6)	12 (22.6)	0.991	45 (22.8)	3 (42.9)	0.252
Class 2	17 (6.6)	1 (1.9)	0.180	12 (6.1)	1 (14.3)	0.485
Class 3	13 (5.1)	1 (1.9)	0.311	12 (6.1)	0 (0)	0.421
Hypertension	138 (53.7)	31 (58.5)	0.523	95 (47)	4 (57.1)	0.568
Diabetes	52 (20.2)	8 (15.1)	0.389	39 (19.3)	0 (0)	0.205
IFG	21 (8.2)	7 (13.2)	0.289	14 (6.9)	3 (42.9)	0.001
IGT	9 (3.5)	3 (5.7)	0.438	5 (2.5)	1 (14.3)	0.071
Dyslipidemia	119 (46.3)	22 (41.5)	0.523	88 (43.6)	2 (28.6)	0.444
Stroke	8 (3.1)	0 (0)	0.359	4 (2.0)	1 (14.3)	0.039
MI	9 (3.5)	0 (0)	0.366	7 (3.5)	0 (0)	0.612
ATS	7 (2.7)	4 (7.5)	0.099	4 (2.0)	1 (14.3)	0.039
AH use	128 (49.8)	28 (52.8)	0.688	87 (43.1)	4 (57.1)	0.468
AHA use	41 (16)	5 (9.4)	0.224	34 (16.8)	0 (0)	0.601
Statin use	51 (19.8)	5 (9.4)	0.073	34 (16.8)	0 (0)	0.603

Data are expressed as mean (SD), median (IQR), or absolute number (percentage). AI enlargement+: patients with AI showing significant mass enlargement during follow up. AI enlargement−: patients with AI showing not significant mass enlargement during follow up. ACSfu+: patients with AI developing ACS during follow-up. ACSfu−: patients with AI not developing ACS during follow-up. SI conversion factors: cortisol, ×27.59; ACTH, ×0.22; total and HDL cholesterol, ×0.026; triglicerides, ×0.011; and FPG, ×0.06

*ACS* autonomous cortisol secretion, *AH* antihypertensive, *AHA* antihyperglycemic agent, *ATS* atherosclerosis, *DBP* diastolic blood pressure, *FPG* fasting plasma glucose, *HDL-C* high-density lipoprotein cholesterol, *IFG* impaired fasting glucose, *IGT* impaired glucose tolerance, *LDL-C* low-density lipoprotein cholesterol, *MI* myocardial infarction, *SBP* systolic blood pressure

### Risk of mass enlargement during follow-up

**Fig. 4:**
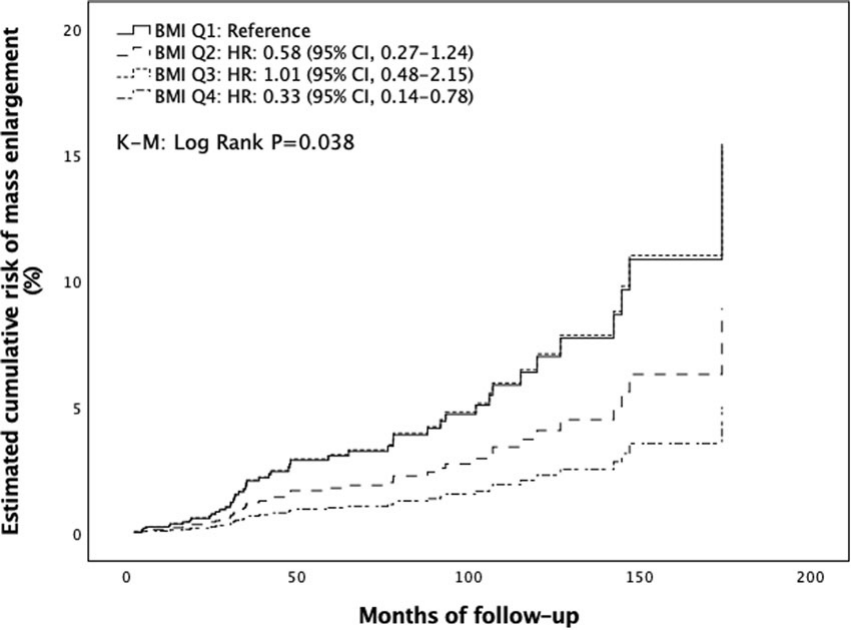
Estimated cumulative risk of adrenal mass enlargement during follow-up according to BMI quartiles

Overall, 53 patients out of 310 (17.1%) showed significant dimensional growth of adenoma during follow-up. The cumulative risk for mass enlargement was 8.4%, 10.3%, and 10.9% after 2, 3, and 5 years, respectively. Patients who experienced an increase in mass size during follow-up showed a higher frequency of ACS (39.6 vs. 23.3%, *P* = 0.014) and lower prevalence of NFAIs (49.1 vs. 71.2%, *P* = 0.002) at diagnosis compared to those without significant enlargement of adrenal lesions (Supplementary Material S5). However, the survival analysis showed no difference in the risk of mass enlargement between NFAI and ACS patients (K–M log rank: 1.167, *P* = 0.280) (Supplementary Material S6). Furthermore, those showing a significant adrenal mass growth had lower BMI (26.8 ± 4.9 vs 28.8 ± 5.8 kg/m^2^, *P* = 0.022) and higher post 1 mg DST cortisol levels (1.7 [1.1–2.7] vs 1.3 [0.9–2.3] μg/dl, *P* = 0.047), compared to those with an unchanged size of mass during follow-up (Table 4). Stratifying for quartiles of BMI (Q1, BMI ≤ 24.57 kg/m^2^; Q2, BMI 24.58–28.08 kg/m^2^; Q3, BMI 28.09–31.24 kg/m^2^; Q4, BMI > 31.24 kg/m^2^), the incidence of adrenal mass enlargement was 5.0/1000 PYs, 3.3/1000 PYs, 4.5/1000 PYs, and 1.7/1000 PYs, for Q1, Q2, Q3, and Q4, respectively. The increase in BMI was associated with a lower risk of mass enlargement (K–M log rank: 8.416, *P* = 0.038). After adjusting for multiple confounders, the risk of mass enlargement was significantly lower in subjects in Q4 than those in Q1 (HR 0.33; 95% CI, 0.14–0.78; *P* = 0.012) (Fig. 4) (Supplementary Material S7). Only one patient diagnosed as having a 6 cm adrenal mass and who initially denied the adrenalectomy procedure, showed clinical features of hypercortisolism and conspicuous tumor growth (from 6 to 13 cm) over 12 months. The patient was then referred to surgery; the histological examination revealed findings consistent with adrenocortical cancer. It is worth noting that none of the adrenal tumors initially determined to be benign transformed into adrenocortical carcinoma during follow-up.

## Discussion

In the present study, we evaluated the risk of development of endocrine hyperfunction and mass enlargement during follow-up in a large cohort of patients with AIs, which included both NFAIs and hormone-secreting adrenal lesions. Available data regarding the prevalence of ACS, risk of adrenal mass enlargement, and hormone secretion in patients with AIs are controversial, probably on account of wide methodological heterogeneity of several studies so far reported. In agreement with other series [4, 8, 21, 22], we found that the majority of AIs (67.4%) were NFAIs, with ACS being the most frequent hormonal abnormality found at diagnosis (26.1%). An increased frequency of cardiovascular risk factors, including obesity, dyslipidemia, hypertension, and glucose intolerance has been reported in patients with AIs [23], especially in those with ACS [8, 13, 14, 24, 25]. Thus, hormone excess and, in particular, hypercortisolism, even mild, may result in a notable risk for cardiometabolic morbidity and mortality. In contrast, we found no differences in lipid profile and prevalence of mild glucose impairment or DM between NFAI and ACS patients. Hypertension affected more than half of the patients at diagnosis. It was more frequent in ACS compared to NFAI patients, independently from age, sex, and BMI, as previously demonstrated [13, 26]. In agreement with previous reports [27–29], age, tumor size, and bilateral AIs were the only parameters independently associated with the presence of ACS at diagnosis. Out of 209 patients with NFAI, 3 (1.4%) developed overt endocrine function over time: one developed PHA, while two pheochromocytoma, one of which complicated by Takotsubo syndrome as the first manifestation of acute catecholamine surge [30]. Only two patients with ACS (2.5%) developed CS during follow-up, confirming that ACS is not a condition at high risk for the development of overt hypercortisolism [2, 31–35]. The new development of overt hormone secretion is an extremely rare event in the natural history of AIs, with an estimated pooled risk below 0.3% [15, 18, 36]. CS is the most common clinically apparent endocrine abnormality found during the follow-up in NFAIs, while only three cases of pheochromocytoma and no aldosterone-secreting adenomas have been described to date [26]. It is worth noting that 83% of the subjects with incident overt hormone secretion showed a concomitant enlargement of the adrenal mass, in agreement with previously reported data [37]. Two out of three patients with NFAI developed a new overt endocrine hyperfunction after 2 and 3 years from the diagnosis, while one showed a new apparent catecholamine production after 6 years. These findings are in keeping with currently available data, showing a peak of risk of new endocrine secretion within 3–4 years after the initial diagnosis of AI, and highlight the need, in particular circumstances, for a longer follow-up, to promptly detect the development of adrenal hyperfunction [36]. We observed that seven (3.3%) patients with NFAIs at diagnosis developed ACS over time, in agreement with previous data showing a risk of ACS development up to 12% after a mean follow-up period of 3 years [2, 31, 38–41]. In accordance with some [32, 42], but not all authors [31, 34], none of the patients initially diagnosed as having ACS showed a normalization of the adrenal function during the follow-up. Several studies report AI size as an important predictor of endocrine function development over time, and it has been suggested that AIs ≥3 cm are more likely to develop subtle hypercortisolism than smaller tumors [22, 35]. In the present study, we found that patients with adenoma ≥28 mm had more than tenfold increased risk of developing ACS during the follow-up period compared to those with smaller adrenal lesions. Furthermore, ACS was more prevalent in bilateral incidentalomas, an observation that others have also reported [27, 28], and that has been confirmed by two different metanalyses [26, 43]. Nonetheless, patients with bilateral AIs had not only an increased prevalence but also a significantly higher incidence rate of newly diagnosed ACS during follow-up compared to those with unilateral adrenal lesions. However, consistently with some of previously published studies [28, 29] but not with others [43], risk of ACS development was not independent of adenoma size, providing evidence that it is rather the mass size itself than the bilaterality, which is responsible for the higher frequency of ACS [44]. Patients who developed ACS showed, at baseline, significantly lower levels of ACTH and higher prevalence of glucose intolerance, stroke, and atherosclerosis than those not showing ACS over time. Hence, it could be speculated that these adenomas are characterized by such a low degree of cortisol secretion that goes undiagnosed trough the currently available tests. Moreover, since hypercortisolism is known to be linked to metabolic derangement and related complications [14], this could explain the higher prevalence of cardiovascular risk factors found at study entry in patients with NFAI who developed ACS during follow-up. All these findings suggest that, even in the absence of a clear diagnosis of ACS, the presence of low/suppressed ACTH levels, particularly if associated with AIs of greater size, is important for planning an adequate follow-up. Tumor enlargement has been reported in patients with AIs [2, 23, 32, 45]. Overall, in our cohort, 17% of patients showed a dimensional growth of adenoma during follow-up, with a low cumulative risk (~10%) after 5 years. Interestingly, we found that those in the lower quartile of BMI had a higher risk of significant mass growth during follow-up. The existence of an adipose–adrenal axis is well known [46]; however, no studies have compared the risk of adrenal mass growth in different BMI categories, and few authors have assessed the direct and indirect effect of adipose tissue on adrenal growth. In an in vitro study, Paschke et al. reported that adiponectin receptors were present in all layers of the rat adrenal cortex and medulla, and treatment with adiponectin enhanced adrenocortical cell proliferation [47]. Since adiponectin secretion is impaired in obesity [48, 49], this might explain the inverse relationship between BMI and the risk of mass enlargement. By contrast, leptin was seen to downregulate the expression of several steroidogenic enzymes [50, 51], while other studies [52] have shown a leptin-dependent suppression of HPA activity. Finally, Wnt/beta-catenin, which is one of the major genes involved in adrenal tumorigenesis, is downregulated by obesity-induced low-grade inflammation trough the upregulation of FOXO [53–56]. Altogether, these data might explain the relationship between body mass and risk of adrenal mass enlargement found in the present series. However, further specifically designed studies are warranted to shed some lights on this association. Summarizing, we have evaluated the risk of newly diagnosed hormone secretion and change in adenoma size over the time in a large cohort of patients with AI, considering the effective time of follow-up and adjusting for multiple potential confounders. However, the present study also has some significant limitations that should be considered. The small number of patients developing ACS during follow-up might have affected the validity of our observations. At the same time, the retrospective design of the study cannot allow establishing cause and effect relationship. Finally, patients included in this study were referred to a tertiary care university medical center, which may be associated with selection bias in that this cohort does not represent the overall population of patients with AIs. In conclusion, our results show that AIs have a low risk of morphologic and functional modification over the time, although some clinical, biochemical, and radiological characteristics might identify patients at higher risk for mass enlargement and development of hormonal hyperfunction. ACS is the most common endocrine abnormality found during follow-up and is associated with a higher degree of metabolic impairment. Adenomas ≥ 28 mm, bilaterally located tumors, or presence of low ACTH values at diagnosis were found to be associated with newly diagnosed ACS over time, so a long-term follow-up may be recommended in AI patients with these characteristics. A nonnegligible number of AIs show a growth tendency, especially in individuals with a lower BMI, and mass enlargement can be associated with the development of overt hyperfunction. Hence, a holistic approach taking account of multiple aspects of patients with AIs is warranted for planning a tailored follow-up.

## Supplementary information

Supplementary Materials
