# Supplementary material for: Clinical features, risk of mass enlargement, and development of endocrine hyperfunction in patients with adrenal incidentalomas: a long-term follow-up study
Source: Endocrine. 2020 Sep 11;71(1):178–88. doi: 10.1007/s12020-020-02476-1 (PMC7835305; doi:10.1007/s12020-020-02476-1)
Supplement: Supplementary file 1 — Supplementary Materials [file 12020_2020_2476_MOESM1_ESM.docx]

**Supplementary material**

**Article title:** Clinical features, risk of mass enlargement and development of endocrine hyperfunction in patients with adrenal incidentalomas: a long-term follow-up study

**Journal name:** Endocrine

**Author names:** Pierpaolo Falcetta, Francesca Orsolini, Elena Benelli, Patrizia Agretti, Paolo Vitti, Caterina Di Cosmo, Massimo Tonacchera

**Affiliation and e-mail address of the corresponding author:** Department of Clinical and Experimental Medicine, Section of Endocrinology, University Hospital of Pisa.

Via Paradisa, 2. 56124, Pisa, Italy

Falcetta.pierpaolo@gmail.com

**S1** ROC curve analysis for adenoma size in predicting the occurrence of incident ACS development


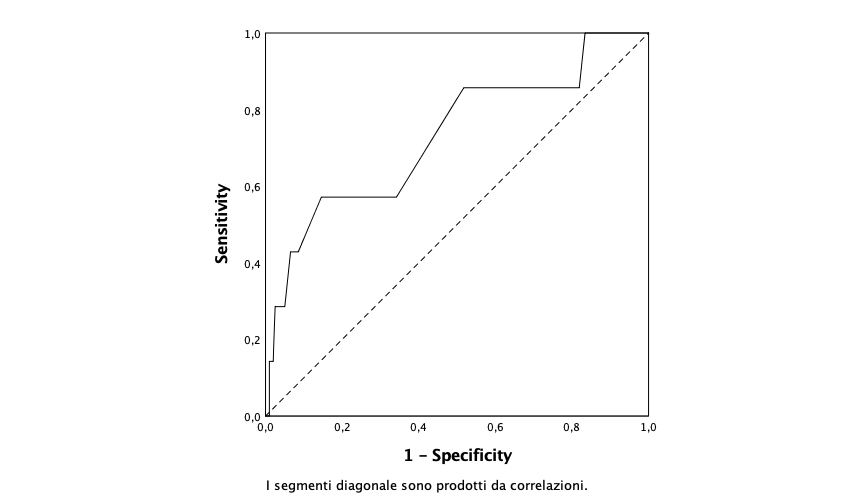


**AUC: 0.730 (95 CI, 0.517-0.943); P=0.039**

**28 mm**

**S2** Cox proportional hazard model for risk of ACS development during follow-up according to AI size

|  | **HR** | **95% C.I.** | **P value** |
| --- | --- | --- | --- |
| **Age, 1 year** | 0.99 | 0.92-1.08 | 0.990 |
| **Gender, male** | 1.07 | 0.19-6.01 | 0.935 |
| **BMI, 1 kg/m^2^** | 0.95 | 0.83-1.08 | 0.433 |
| **AI size > 28 mm** | 12.46 | 2.33-66.52 | 0.003 |

AI, adrenal incidentaloma; BMI, body mass index.

**S3** Cox proportional hazard model for risk of ACS development during follow-up according to localization of AI

|  | **HR** | **95% C.I.** | **P value** |
| --- | --- | --- | --- |
| **Model 1** |  |  |  |
| **Age, 1 year** | 1.02 | 0.94-1.10 | 0.705 |
| **Gender, male** | 0.82 | 0.15-4.38 | 0.815 |
| **BMI, 1 kg/m^2^** | 0.98 | 0.86-1.13 | 0.811 |
| **Bilateral AI** | 5.36 | 1.17-24.48 | 0.030 |
| **Model 2** |  |  |  |
| **Age, 1 year** | 0.99 | 0.92-1.08 | 0.938 |
| **Gender, male** | 1.31 | 0.22-7.70 | 0.765 |
| **BMI, 1 kg/m^2^** | 0.98 | 0.85-1.12 | 0.747 |
| **Bilateral AI** | 3.89 | 0.83-18.32 | 0.085 |
| **Mass size, 1 mm** | 1.11 | 1.01-1.21 | 0.022 |

Model 1: adjusted for age, sex, size, and BMI. Model 2: adjusted for all the variables included in Model 1 + “Mass size”

AI, adrenal incidentaloma; BMI, body mass index.

**S4** Cox proportional hazard model for risk of ACS development during follow-up according to levels of ACTH at entry

|  | **HR** | **95% C.I.** | **P value** |
| --- | --- | --- | --- |
| **Age, 1 year** | 1.02 | 0.93-1.12 | 0.638 |
| **Gender, male** | 0.92 | 0.16-5.12 | 0.922 |
| **BMI, 1 kg/m^2^** | 1.00 | 0.86-1.17 | 0.956 |
| **AI size** | 1.13 | 1.03-1.23 | 0.011 |
| **ACTH < 10 pg/ml** | 11.2 | 2.06-60.77 | 0.005 |

AI, adrenal incidentaloma; BMI, body mass index.

**S5** Frequency of endocrine abnormalities at baseline in patients showing adrenal mass enlargement during the follow-up

|  | **AI enlargement –**  **(n=257)** | **AI enlargement +**  **(n=53)** | **P value** |
| --- | --- | --- | --- |
| **NFAA** | 183 (71.3) | 26 (49.1) | 0.002 |
| **ACS** | 60 (23.3) | 21 (39.6) | 0.014 |
| **Cushing’s Syndrome** | 7 (2.7) | 2 (3.8) | 0.654 |
| **Hyperaldosteronism** | 6 (2.3) | 3 (5.6) | 0.099 |
| **Pheochromocytoma** | 1 (0.4) | 1 (1.9) | 0.313 |

Data are expressed as absolute number (percentage). ACS, autonomous cortisol secretion; AI, adrenal incidentaloma; AI enlargement -, patients with AI who dNFAA, non-functioning adrenal incidentaloma.

**S6** Cumulative risk of adrenal mass enlargement during follow-up according to presence of ACS at diagnosis

**
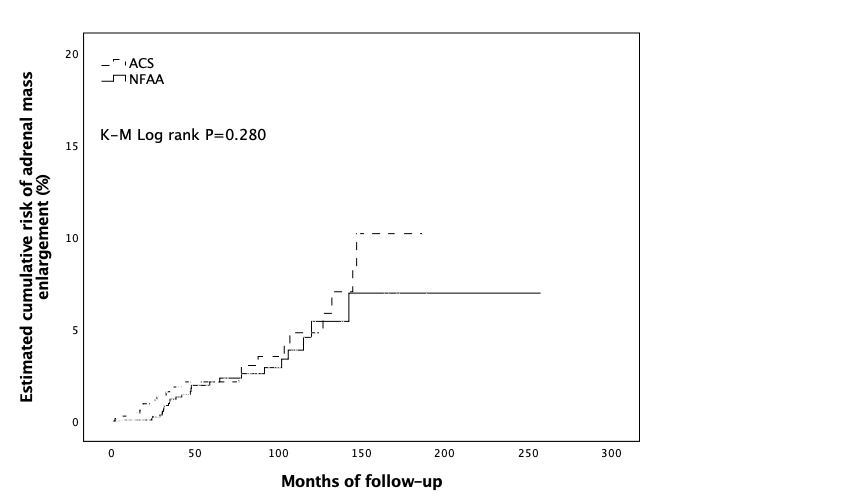
**

**S7** Cox proportional hazard model for risk of mass enlargement during follow-up according to BMI categories

|  | **HR** | **95% C.I.** | **P value** |
| --- | --- | --- | --- |
| **Age, 1 year** | 1.00 | 0.98-1.03 | 0.614 |
| **Gender, male** | 0.77 | 0.41-1.45 | 0.430 |
| **AI size, 1 mm** | 1.00 | 0.97-1.03 | 0.828 |
| **BMI quartiles**  **Q1**  **Q2**  **Q3**  **Q4** | Ref  0.58  1.015  0.33 | Ref  0.27-1.24  0.48-2.15  0.14-0.78 | Ref  0.162  0.970  0.012 |

AI, adrenal incidentaloma; BMI, body mass index.
